# Complete Genome Sequence of Hyperthermophilic Piezophilic Archaeon *Palaeococcus pacificus* DY20341^T^, Isolated from Deep-Sea Hydrothermal Sediments

**DOI:** 10.1128/genomeA.01080-15

**Published:** 2015-09-17

**Authors:** Xiang Zeng, Mohamed Jebbar, Zongze Shao

**Affiliations:** a Key Laboratory of Marine Biogenetic Resources, Third Institute of Oceanography, State Oceanic Administration (SOA), Xiamen, People’s Republic of China; b Key Laboratory of Marine Genetic Resources of Fujian Province, Xiamen, Fujian, People’s Republic of China; c South China Sea Bio-Resource Exploitation and Utilization Collaborative Innovation Center, Xiamen, Fujian, People’s Republic of China; d Université de Bretagne Occidentale (UBO, UEB), Institut Universitaire Européen de la Mer (IUEM)–UMR 6197, Laboratoire de Microbiologie des Environnements Extrêmes (LM2E), Plouzané, France; e CNRS, IUEM–UMR 6197, Laboratoire de Microbiologie des Environnements Extrêmes (LM2E), Plouzané, France; f Ifremer, UMR6197, Laboratoire de Microbiologie des Environnements Extrêmes (LM2E), Plouzané, France

## Abstract

We report the genome sequence of *Palaeococcus pacificus* DY20341^T^, isolated from a sediment sample collected from eastern Pacific Ocean hydrothermal fields, which is the first report of a complete genome for a *Palaeococcus* species. The genome sequence will help to better understand differentiation phylogenetic relationships and evolution of several *Thermococcales* species.

## GENOME ANNOUNCEMENT

The order *Thermococcales* is one of the best-studied groups of hyperthermophiles. Over the past 20 years, ~40 species affiliated with three genera (32 *Thermococcus*, 5 *Pyrococcus*, and 3 *Palaeococcus*) have been described in details. The comparative genome analysis results showed that they display a characteristic high level of rearrangements (1). *Palaeococcus pacificus* DY20341^T^ is a hyperthermophilic piezophilic anaerobic archaeon that was isolated from a sediment sample collected from eastern Pacific Ocean hydrothermal fields (S 1.37° W 102.45°) at a depth of 2,737 m. The *Pa. pacificus* can grow at temperatures ranging from 50°C to 90°C (optimally at 80°C) and optimally under 30 MPa, showing typical hyperthermophilic and piezophilic features. Phylogenetic analysis based on 16S rRNA gene sequences showed that it was most closely related to *Palaeococcus ferrophilus*, with a similarity of 95.7% (2).

Pyrosequencing was performed on a Roche 454 GS-FLX Titanium system by Shanghai Hanyu Bio-Tech Company. The contigs were ordered using the Newbler assembler (Roche Diagnostics, Basel, Switzerland), and gap filling and closure was achieved by combinatorial multiplex PCR and sequencing. The genome of *Pa. pacificus* DY20341^T^ is a single circular 1,859,370-bp chromosome without an extrachromosomal element, with a G+C content of 43.04%. A total of 2,001 protein-coding sequences were predicted by Glimmer version 3.02. Of the genes, 78.11%  (1,563/2,001) were assigned to specific Clusters of Orthologous Groups (COG) Database functional gene groups, and 49.83% (997/2,001) were assigned an enzyme classiﬁcation number. The average gene size is 845 bp, comprising CDSs ranging from 38 to 1,749 amino acids. tRNA genes were predicted using tRNAscan-SE 1.21 and ARAGORN, rRNA genes using RNAmmer version 1.2. Two rRNA loci, and 47 tRNA genes were detected. Three detected tRNA genes with C-loop introns are tRNA-Lys(ctt) with 1461 bp intron; tRNA-Ser(aga) with 29 bp intron; tRNA-Trp(cca) with 71 bp intron.

Similar with other *Thermococcales* species, *Pa. pacificus* possesses an incomplete tricarboxylic acid (TCA) cycle, a modified Embden-Meyerhof pathway to glycolysis and metabolism pathway of proteins and carbohydrates (3–7). The conserved respiration system is also represented by membrane-bound hydrogenase and A_0_A_1_-type ATP synthase (8).

Different with *Pyrococcus* species, *Pa. pacificus* does not contain “saccharolytic gene island” and “maltose and trehalose degradation gene” (5), which carries a set of genes responsible for the utilization of cellulose, laminarin, agar, maltose, trehalose, and other β-linked polysaccharides. These genes are also absent in *Thermococcus* species except *Thermococcus sibiricus* (5).

Analysis of the repeated sequences and a search against the IS database revealed that a remarkable feature of the *Pa. pacificus* genome is the absence of mobile genetic elements including transposons, transposases, integrases and virus-related region. It indicates that *Pa. pacificus* genome has not been subjected to frequent or recent genomic rearrangements during its evolution.

Further comparative genomic studies of several *Thermococcales* species will provide a foundation to identify the biochemical mechanisms responsible for its adaptation to a hydrothermal vent environment and to understand the systematic evolution of members in this order.

### Nucleotide sequence accession number.

This whole-genome shotgun project has been deposited in DDBJ/ENA/GenBank under the accession number CP006019.

## References

[1] Cossu M, Da Cunha V, Toffano-Nioche C, Forterre P, Oberto J 2015 Comparative genomics reveals conserved positioning of essential genomic clusters in highly rearranged *Thermococcales* chromosomes. Biochimie [Epub ahead of print.] doi:10.1016/j.biochi.2015.07.008. PMC4640148 26166067

[2] Zeng X, Zhang X, Jiang L, Alain K, Jebbar M, Shao Z 2013 *Palaeococcus pacificus* sp. nov., an archaeon from deep-sea hydrothermal sediment. Int J Syst Evol Microbiol 63:2155–2159. doi:10.1099/ijs.0.044487-0. 23104364

[3] Fukui T, Atomi H, Kanai T, Matsumi R, Fujiwara S, Imanaka T 2005 Complete genome sequence of the hyperthermophilic archaeon *Thermococcus kodakaraensis* KOD1 and comparison with *Pyrococcus* genomes. Genome Res 15:352–363. doi:10.1101/gr.3003105. 15710748 PMC551561

[4] Bridger SL, Lancaster WA, Poole FL, Schut GJ, Adams MW 2012 Genome sequencing of a genetically tractable *Pyrococcus furiosus* strain reveals a highly dynamic genome. J Bacteriol 194:4097–4106. doi:10.1128/JB.00439-12. 22636780 PMC3416535

[5] Mardanov AV, Ravin NV, Svetlitchnyi VA, Beletsky AV, Miroshnichenko ML, Bonch-Osmolovskaya EA, Skryabin KG 2009 Metabolic versatility and indigenous origin of the archaeon *Thermococcus sibiricus*, isolated from a Siberian oil reservoir, as revealed by genome analysis. Appl Environ Microbiol 75:4580–4588. doi:10.1128/AEM.00718-09. 19447963 PMC2704819

[6] Lee HS, Kang SG, Bae SS, Lim JK, Cho Y, Kim YJ, Jeon JH, Cha SS, Kwon KK, Kim HT, Park CJ, Lee HW, Kim SI, Chun J, Colwell RR, Kim SJ, Lee JH 2008 The complete genome sequence of *Thermococcus onnurineus* NA1 reveals a mixed heterotrophic and carboxydotrophic metabolism. J Bacteriol 190:7491–7499. doi:10.1128/JB.00746-08. 18790866 PMC2576655

[7] Zivanovic Y, Armengaud J, Lagorce A, Leplat C, Guérin P, Dutertre M, Anthouard V, Forterre P, Wincker P, Confalonieri F 2009 Genome analysis and genome-wide proteomics of *Thermococcus gammatolerans*, the most radioresistant organism known amongst the *Archaea*. Genome Biol 10:R70. doi:10.1186/gb-2009-10-6-r70. 19558674 PMC2718504

[8] Schut GJ, Boyd ES, Peters JW, Adams MW 2013 The modular respiratory complexes involved in hydrogen and sulfur metabolism by heterotrophic hyperthermophilic archaea and their evolutionary implications. FEMS Microbiol Rev 37:182–203. doi:10.1111/j.1574-6976.2012.00346.x. 22713092

